# Tolerance for corruption and descriptive social norm: An experimental study of embezzlement

**DOI:** 10.1371/journal.pone.0303558

**Published:** 2024-05-20

**Authors:** Sen Tian, Liangfo Zhao

**Affiliations:** China Center for Behavioral Economics and Finance, Southwestern University of Finance and Economics, Chengdu, Sichuan, China; Beijing Normal University, CHINA

## Abstract

Public tolerance for corruption within a society significantly influences the prevalence of corrupt practices, but less is known about how this tolerance evolves with social norms. This paper presents experimental evidences demonstrating that the descriptive social norm indicating widespread corruption can lead to increased tolerance for corruptive acts. We introduce an asymmetric information ultimatum game to simulate the interactions between embezzlers and citizens. Game theoretical analysis reveals that victims anticipating corruption will exhibit greater compliance with embezzlement when the offers are evaluated based on descriptive norms. To test the hypothesis, we employ a framing effect to induce variations in descriptive norms within a behavioral experiment. Although the treatment effect is significant only in the subgroup of student cadres, this subgroup demonstrated increased beliefs about embezzlement, greater tolerance for corruption, and a heightened propensity to embezzle when exposed to framings with hierarchical implications. This paper contributes to the corruption literature by examining the effects of descriptive norms on victims’ responses to embezzlement. It offers a more comprehensive perspective on how social standards shape public opinions and corrupt actions, enhancing our understanding of the self-reinforcing nature of corruption.

## Introduction

Social norms play a crucial role in shaping corruption decisions. The likelihood of individuals engaging in corrupt acts is often influenced by their perceptions of others’ behaviors. Experimental evidence suggests that decision-makers are more prone to accept bribes if they believe that “everyone else is doing it” [1,2]. Beyond those directly involved in corrupt transactions, descriptive norms may also impact civilians who suffer from the negative externalities of corruption. Their attitude toward corruptive acts significantly influences levels of corruption, not only by affecting the moral cost and social pressure faced by perpetrators but also because the victims of corruption can impose sanctions through measures like tip-offs, filing lawsuits, and whistleblowing. Nevertheless, relatively limited attention has been paid to the effect of social norms on the responses of those victimized by corruption. It remains unclear whether social norms associated with the prevalence of corruption will increase or decrease the victims’ tolerance of corruption.

This paper explores the question by studying an embezzlement scenario with direct interaction between embezzlers and victims. Embezzlement is a prevalent form of corruption within hierarchical political systems [3,4], characterized by a top-down distribution of political power and resources [5,6]. In Parallel with the bribery games discussed in the literature, embezzlement also plays a crucial role in bureaucratic corruption in developing economies, typically involving officials misappropriating public resources [7,8].

Existing experimental studies on corruption frequently utilize the framework of the bribery game, which concentrates on the interactions between bribers and officials [9–11]. In these frameworks, victims of corruption typically play a passive role. Our study aims to investigate the decisions of citizens, who are both legitimate owners of public resources and the victims of embezzlement. Therefore, we incorporate citizens’ ability to punish embezzlers into our model. We introduce an ultimatum game with asymmetric information, wherein a monetary endowment is owned equally by both a proposer and a responder. The endowment is subject to a potential random shock, which may reduce its value. If there is no shock, the proposer must choose one of the two options to allocate the endowment: equal division or embezzlement. The responder’s payoff from the embezzlement scenario is the same as that received after the shock, so the responder cannot tell whether the shock happened. This information asymmetry mimics the opacity common to corrupt activities, typically out of public view. The responder must then decide to either accept or reject the proposer’s offer, with rejection resulting in no payoff for either party. In this game, the proposer symbolizes an official with the power to distribute resources, while the responder represents the citizens who are impacted by their actions. The punishment option models the importance of public approval in governance systems [12,13].

Our study’s primary focus is on the impact of descriptive norms on responders’ decisions. The descriptive norm indicates the perceived frequency of a specific action [14]. Within the embezzlement game, we concentrate on the descriptive norm of corruption, indicated by the perceived likelihood of proposers opting to embezzle. Compared to injunctive norms, descriptive norms are more closely connected to actual decisions and more malleable [15], therefore more appropriate for examining the evolution of corruption norms. Regarding how responders react to the descriptive norm of embezzlement, there exist two opposing predictions. The first is derived from theories that regard equity as the benchmark for fairness (e.g., inequality aversion theory and ERC model [16,17]). The equity-based theory suggests that as responders believe proposers are more likely to embezzle, they may perceive greater unfairness and, therefore, are more willing to reject the low offer as a form of punishment. The second prediction derives from a norm-based fairness preference model, in which we hypothesize that fairness is evaluated based on social norms instead of equity. This model predicts that when corruption is widespread and thus becomes a social norm, people may become more tolerant of inequality, as the corrupt norm effectively lowers the fairness benchmark, reducing the felt disutility from a lesser offer. The equity-based model implies that the spread of corruption is self-limiting, with victims’ propensity to punish increasing as corruption intensifies. Conversely, the norm-based model suggests that corruption might engender a self-perpetuating cycle, where current corruption leads to future tolerance and, thereby, even more corruption.

In an experiment replicating the design of the embezzlement game, we introduced variations in game framing to induce exogenous shifts in the descriptive norm of corruption. Specifically, in the hierarchical treatment, proposers were labeled as “officials” and responders as “commoners”, while in the baseline treatment, they were referred to as Participant A and Participant B. This study was conducted in China, a society with a long-standing hierarchical culture in history, so we expect players’ perceived descriptive norm of corruption will be influenced by the implied status of the titles. The norm-based hypothesis predicts that any change in responders’ beliefs about embezzlement across treatments will be positively correlated with their acceptance rate of lower offers. Conversely, the equity-based hypothesis predicts a negative correlation.

The experimental results align with the norm-based hypothesis. Within the subgroup of student cadres, the hierarchical framing significantly increased both responders’ perceived norm of corruption and their acceptance rate of lower offers. Student cadres refer to students recruited by Universities to support the college administration in managing their peers. When the student cadres acted as proposers, they exhibited weakly higher belief in the likelihood of responders accepting low offers and were more inclined to embezzle when presented with hierarchical framing. The cadre group consistently displayed an increased propensity for embezzlement and a higher tolerance for corruption within the hierarchical context.

This paper is closely related to the experimental literature on social norms and corruption. Prior research has established that the presence of descriptive norms suggesting widespread corruption can encourage bribery practices [1,18,19], driven by mechanisms such as diminished guilt aversion [20], parochial altruism [21], and direct reciprocity [22]. While the majority of these studies have focused on the perpetrators of corruption, such as bribe-takers and givers, comparatively less attention has been paid to the impact of descriptive norms on the victims’ decision-making processes. In contrast to research that examines norms and corruption from the perspective of the perpetrators, our study explores the influence through the perspective of social compliance. We propose that the eroding norms may lead to increased victim tolerance for corrupt acts, potentially resulting in an escalation of such behaviors. Among the several experimental studies that consider corruption victims, Salmon and Serra [23] investigated how cultural differences affect victims’ judgments of perpetrators; Makowsky and Wang [24] used a sequential embezzlement game where players are both embezzlers and victims to study how the organization architecture influences corruption; Guerra and Zhuravleva [25] looked at the willingness of neutral bystanders to punish bribery; Abbink et al. [26] discussed the policy tools to combat corruption where victims may be harassed to paying bribes; He and Jiang [27] analyzed the influence of partisan culture and identity on decisions within the harassment bribery framework. Our research distinguishes itself by emphasizing the role of descriptive norms and considering victims’ capability to impose significant penalties on corrupt behavior.

The remainder of this paper is organized as follows: The section on materials and methods outlines our initial hypotheses derived from theoretical analysis, followed by a detailed account of the experimental design. Findings from the study are presented in the results section. Subsequently, the discussion section offers insights into the implications of these findings and possible extensions for future research.

## Materials and methods

This section reports on the research methods in two parts. Initially, we present a simple model to examine the decisions of an embezzler and a citizen, which leads to the formulation of testable hypotheses. In the second part, we introduce the design and procedure of the experiment.

### Theoretical hypotheses

In the embezzlement game, our primary interest lies in the impact of descriptive norms on a victim’s response to corruption. To elucidate this mechanism, we propose a norm-based fairness preference model that considers both monetary payoff and the psychological utility derived from perceptions of fairness, as fairness is intrinsically linked to descriptive norms [28–32].

Consider a game involving 2 subjects, indexed by *i*∈(1,2), and let *π*_*i*_(*s*_*i*_,*s*_*j*_) denote the monetary payoff for player *i* when they choose strategy *S*_*i*_∈*S*_*i*_. Define *p*′(*s*_*j*_) as *i*’s belief on the probability of subject *j* adopting stretegy *s*_*j*_, and $\sum_{s_{j}\in S_{j}}p^{\prime }(s_{j})=1.p^{\prime }(s_{j})$ serves as a proxy for the descriptive norm in this model. Then, the utility function for subject *i* can be expressed as:

$$U(s_{i})=\pi_{i}-\alpha_{i}max(\pi_{i}-\sum_{s_{j}\in S_{j}}\pi_{i}(s_{i},s_{j})p^{\prime }(s_{j}),0)-\beta_{i}max(\sum_{s_{j}\in S_{j}}\pi_{i}(s_{i},s_{j})p^{\prime }(s_{j})-\pi_{i},0)$$ (1)

Here, the term $\pi_{i}-\sum_{s_{j}\in S}\pi_{i}(s_{i},s_{j})p^{\prime }(s_{j})$ represents the discrepancy between the anticipated payoff shaped by the presumed norm and the actual payoff. The parameters *α*_*i*_ and *β*_*i*_ measure the extent of subject *i*’s aversion to this disparity, with $\beta_{i}=\lambda \alpha_{i},0<\alpha_{i}<1,\lambda >1$. Moreover, *α*_*i*_ is private information known only to player *i*. For simplicity, we assume *α*_*i*_ is uniformly distributed over the interval [0,1], leading to a cumulative distribution function *F*(*α*_*i*_) = *α*_*i*_.

According to Eq (1), psychological costs arise when a player’s payoff deviates from the expectations set by social norms, with the costs escalating when the player’s earnings fall below the normative benchmark. Previous models, such as the inequality aversion theory and the ERC model [16,17], adopted equity as the reference point for fairness, so psychological disutility occurs when payoffs are unequal. The norm-based preference suggests that fairness is measured by the deviation from descriptive norms. Consequently, an uneven distribution of payoffs may still be perceived as fair if it aligns with the prevailing norm.

Then, we use a modified ultimatum game to approximate decisions in the embezzlement scenario. In the ultimatum game, the proposer has greater allocation authority than the responder. Like officials, they can exploit this advantage for personal gain despite the equal ownership of the allotted endowment. In addition, embezzlement can be concealed from external scrutiny. To capture this characteristic, we assume the initial endowment $\bar{V}$ will shrink to a lesser value *V* due to a shock with probability *μ*, where $\bar{V}>\underline{V}>0$. The realization of this shock is privately observable only to the proposer, while the responder is aware only of its probabilistic distribution.

If the shock occurs, the reduced endowment will be automatically split into $\left(\frac{\underline{V}}{2},\frac{\underline{V}}{2}\right)$, with each player taking half. If the shock does not occur, the proposer can choose one of the two options: keeping $\bar{V}-\frac{\underline{V}}{2}$ for himself, leaving $\frac{\underline{V}}{2}$ to the responder, or sharing $\bar{V}$ equally. Here, we assume $\bar{V}-\frac{\underline{V}}{2}>\frac{\bar{V}}{2}>\frac{\underline{V}}{2}>0$. Upon observing the proposer’s offer, the responder must choose to accept or reject it. If the responder accepts the offer, each player receives the monetary as specified. If rejected, both players receive zero payoffs.

**Fig 1:**
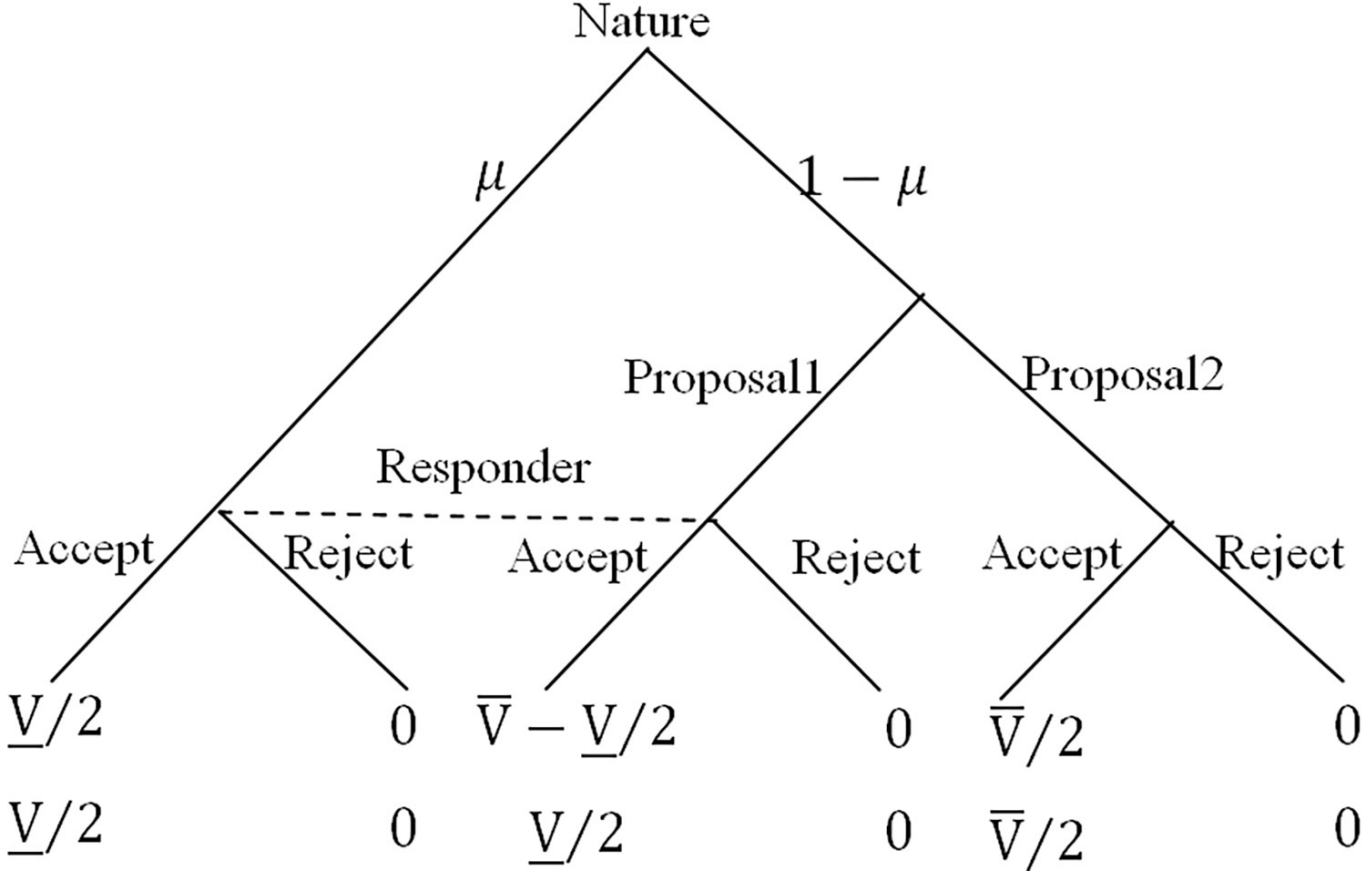
An embezzlement game.

The responder cannot discern whether the low offer $\frac{\underline{V}}{2}$ stems from the shock or the proposer’s embezzlement. Therefore, responders’ decisions rely heavily on their beliefs about the likelihood of embezzlement versus shock occurrence. All the above settings are public information for both players. Fig 1 depicts the game tree:

Consider *p* to be the proportion of proposers choosing “Proposal 1” (overall probability of embezzlement), and *q* represents the fraction of responders who accept $\frac{\underline{V}}{2}$, where *p*,*q*∈[0,1]. Denote *p*′ as the responder’s belief regarding the proposer’s probability of opting to embezzle (first-order belief). *p*′ also reflects the responder’s perceived descriptive norm of corruption. In the event of a shock, the responder’s expected payoff is $\frac{\underline{V}}{2}$. Without the shock, the responder’s expected income is given by $p^{\prime }\frac{\underline{V}}{2}+(1-p^{\prime })\frac{\bar{V}}{2}$. Because the responder cannot observe the shock upon receiving $\frac{\underline{V}}{2}$, the norm-based utility function for the responder can be expressed as:

$$U_{R}(\frac{\underline{V}}{2})=(1-\mu )\left[\frac{\underline{V}}{2}-\beta_{i}(p^{\prime }\frac{\underline{V}}{2}+(1-p^{\prime })\frac{\bar{V}}{2}-\frac{\underline{V}}{2})\right]+\mu \left[(\frac{\underline{V}}{2})\right]$$ (2)

Whether the responder accepts $\frac{\underline{V}}{2}$ depends on the utility difference, $U_{R}(\frac{\underline{V}}{2})-U_{R}(rejection)$. Since rejection indicates punishment against embezzlement, we normalize the psychological utility of punishment to zero so that *U*_*R*_(rejection) = 0. Given that the responder’s *β*_*i*_ is uniformly distributed over [0,*λ*], the proportion of players inclined to accept the low offer when faced with a descriptive norm of corruption at *p*′ can be written as:

$$q(p^{\prime })=\frac{\underline{V}/2}{(1-\mu )\lambda [p^{\prime }\underline{V}/2+(1-p^{\prime })\bar{V}/2]-(\mu +1)\underline{V}/2}$$ (3)

Eq (3) also represents the accumulated probability of receivers accepting the low offer conditional on belief *p*′. This cumulative probability is derived by aggregating individual best response functions across all responders, whose *β*_*i*_ values are distributed uniformly over the interval [0,*λ*]. Accordingly, we will henceforth refer to *q*(*p*′) as the responder’s aggregated best response function. Based on Eq (3), it is straightforward to demonstrate that $\frac{\partial q}{\partial p^{\prime }}=\frac{\underline{V}(\bar{V}-\underline{V})(1-\mu )p^{\prime }}{4{\left((1-\mu )\lambda [p^{\prime }\underline{V}/2+(1-p^{\prime })\bar{V}/2]-(\mu +1)\underline{V}/2\right)}^{2}}>0$. As such, an increase in the responder’s belief about the proposer’s embezzlement correlates with a heightened likelihood of accepting the low offer. When the responder is presented with the high offer $\frac{\bar{V}}{2}$, the expected utility can be calculated as $U_{B_{i}}(\frac{\bar{V}}{2})=\frac{\bar{V}}{2}-\alpha_{i}(\frac{\bar{V}}{2}-p_{i}^{\prime }\frac{\underline{V}}{2}-(1-p_{i}^{\prime })\frac{\bar{V}}{2})$. Given that *α*_*i*_<1, the responder will always accept the high offer.

In examining the behavior of the proposer, let *q*′ denote the proposer *j*’s estimation of the likelihood that the responder will accept the low offer, $\frac{\underline{V}}{2}$. Because proposer *j*’s expected payoff when choosing Proposal 1 is $q^{\prime }(\bar{V}-\frac{\underline{V}}{2})$, Then the proposer *j*’s norm-based utility from embezzlement is expressed as:

$$U_{P}(P1)=q^{\prime }\left[(\bar{V}-\frac{\underline{V}}{2})-\alpha_{j}(\left(\bar{V}-\frac{\underline{V}}{2})-q^{\prime }(\bar{V}-\frac{\underline{V}}{2}\right))\right]+(1-q^{\prime })\left[0-\beta_{j}q^{\prime }(\bar{V}-\frac{\underline{V}}{2})\right]$$ (4)

In addition, since the responder will not reject a high offer, and given that the proposer *j*’s expected payoff from choosing Proposal 2 is $\frac{\bar{V}}{2}$, the expected utility for an equal split is $U_{P}(P2)=\frac{\bar{V}}{2}$. Whether the proposer chooses proposal 1 depends on *U*_*P*_(*P*1)−*U*_*P*_(*P*2), so that the probability of embezzlement is $p=F\left(\alpha_{j}<\frac{(\bar{V}-\underline{V}/2)q^{\prime }-\underline{V}/2}{q^{\prime }(1-q^{\prime })(\bar{V}-\underline{V}/2)(1+\lambda )}\right)$. The proposers’ aggregated best response function is given by:

$$p(q^{\prime })=\frac{(\bar{V}-\underline{V}/2)q^{\prime }-\underline{V}/2}{q^{\prime }(1-q^{\prime })(\bar{V}-\underline{V}/2)(1+\lambda )}$$ (5)

From Eq (5), it is deduced that the probability of embezzlement diminishes as the responder’s rate of rejection increases, i.e., $\frac{\partial p}{\partial q^{\prime }}=\frac{(\bar{V}-\underline{V}/2){\left(q^{\prime }\right)}^{2}-\bar{V}q^{\prime }+\underline{V}/2}{{\left[q^{\prime }(1-q^{\prime })\right]}^{2}(\bar{V}-\underline{V}/2)(1+\lambda )}<0$.

The slope of *q*(*p*′) reflects the responder’s sensitivity to changes in the descriptive norms. The norm-based preference function suggests that the responder is more likely to accept the low offer as the descriptive norm skews towards greater corruption. This finding contrasts with the predictions of models that posit equity as the reference point for fairness. For instance, within the framework of inequality aversion theory, the utility function is specified as follows:

$$U(s_{i})=\pi_{i}(s_{i},s_{j})-\alpha_{i}max(\pi_{i}-\pi_{j},0)-\beta_{i}max(\pi_{j}-\pi_{i},0)$$ (6)

Based on (6), whether the responder accepts the low offer is determined by $U_{R}(\frac{\underline{V}}{2})-U_{R}(rejection)=\frac{(1-\mu )p^{\prime }}{(1-\mu )p^{\prime }+\mu }\left[\frac{\underline{V}}{2}-\beta_{i}(\bar{V}-\underline{V})\right]+\frac{\mu }{(1-\mu )p^{\prime }+\mu }(\frac{\underline{V}}{2})$. Building on the logic established in Eq (3), we derive the aggregated best response function *q* as $q=\frac{(1-\mu )(1-p^{\prime })\mu \underline{V}/2}{\left(1-\mu )p^{\prime }(\bar{V}-\underline{V}\right)}$, where $\frac{\partial q}{\partial p^{\prime }}>0$. This suggests that responders are more inclined to reject the low offer if they perceive it to be the product of embezzlement, which implies a greater degree of inequality. The decision of the proposer to select Proposal 1 is influenced by the difference in utilities $U_{A_{j}}(P1)-U_{A_{j}}(P2)=q^{\prime }\left[(\bar{V}-\frac{\underline{V}}{2})-\alpha_{j}(\bar{V}-\underline{V})\right]-\frac{\bar{V}}{2}$, leading to the aggregated best response function $p=\frac{(\bar{V}-\underline{V}/2)q^{\prime }-\underline{V}/2}{q^{\prime }(\bar{V}-\underline{V})}$, where $\frac{\partial p}{\partial q^{\prime }}<0$. Summarizing the analysis, we can formulate the following hypotheses regarding the decisions of the responder:

**Hypothesis 1a**: According to the norm-based preference model, the responder’s likelihood of accepting the low offer *increases* with the descriptive social norm indicating a higher probability of embezzlement.

**Hypothesis 1b**: According to the equity-based preference model, the responder’s likelihood of accepting low offers *decreases* with the descriptive social norm that indicates a higher probability of embezzlement.

Concerning the proposer’s optimal response function, both models converge on the prediction that $\frac{\partial p}{\partial q^{\prime }}<0$:

**Hypothesis 2**: Proposers’ propensity to engage in embezzlement is positively correlated with their belief about the responder’s acceptance ratio.

The interrelationships posited by these hypotheses can be visualized in the subsequent figure:

The curves depicted in both panels of the figure illustrate the aggregated best response functions for embezzlement (*p*) and acceptance of the low offer (*q*) contingent upon the first-order beliefs *q*′ and *p*′. The intersections of the curves represent the proportion of players choosing *p* or *q* in equilibrium. Given the challenges for players to reach equilibrium in a one-shot experiment, our analysis is principally concerned with the directionality of the slopes of the aggregated best response curves. Panel A is based on the norm-based preference, and Panel B derives from the equity-based preference model. Hypothesis 1a is reflected in the responder’s aggregated best response curve presented in Panel A, whereas the analogous curve in Panel B represents Hypothesis 1b. The two models have opposite predictions about the slope of the curves, thereby offering distinct answers to the central research question: Does a corruptive descriptive norm lead to increased or diminished compliance?

Hypothesis 2 suggests that proposers will be more likely to select Proposal 1 when they expect the low offer to be accepted. Despite the consensus between the two models regarding the prediction for proposers’ aggregated best response function, testing hypothesis 2 remains necessary because it elucidates the channel through which responders’ perceived norms influence proposers’ decisions regarding corruption.

Due to the challenges in directly manipulating descriptive norms in games involving social interactions, we adopted an alternative approach by utilizing the framing effect in the embezzlement game. This method aims to generate exogenous variations in descriptive norms, thereby allowing us to observe if decisions shift in accordance with the norms as predicted by the hypotheses. Framing can significantly alter descriptive norms by influencing how players perceive and react to typical behaviors within the game [33,34]. In our experimental design, the baseline treatment describes participants as Player A and Player B. In contrast, the hierarchical treatment labels proposers as “officials” and responders as “commoners.” Given that the experiment was conducted in China, we expect that hierarchical framing will impact players’ expectations about others differently compared to neutral framing because the hierarchical norms have profoundly shaped the societal views about social hierarchy through the long history of centralized governance.

**Fig 2:**
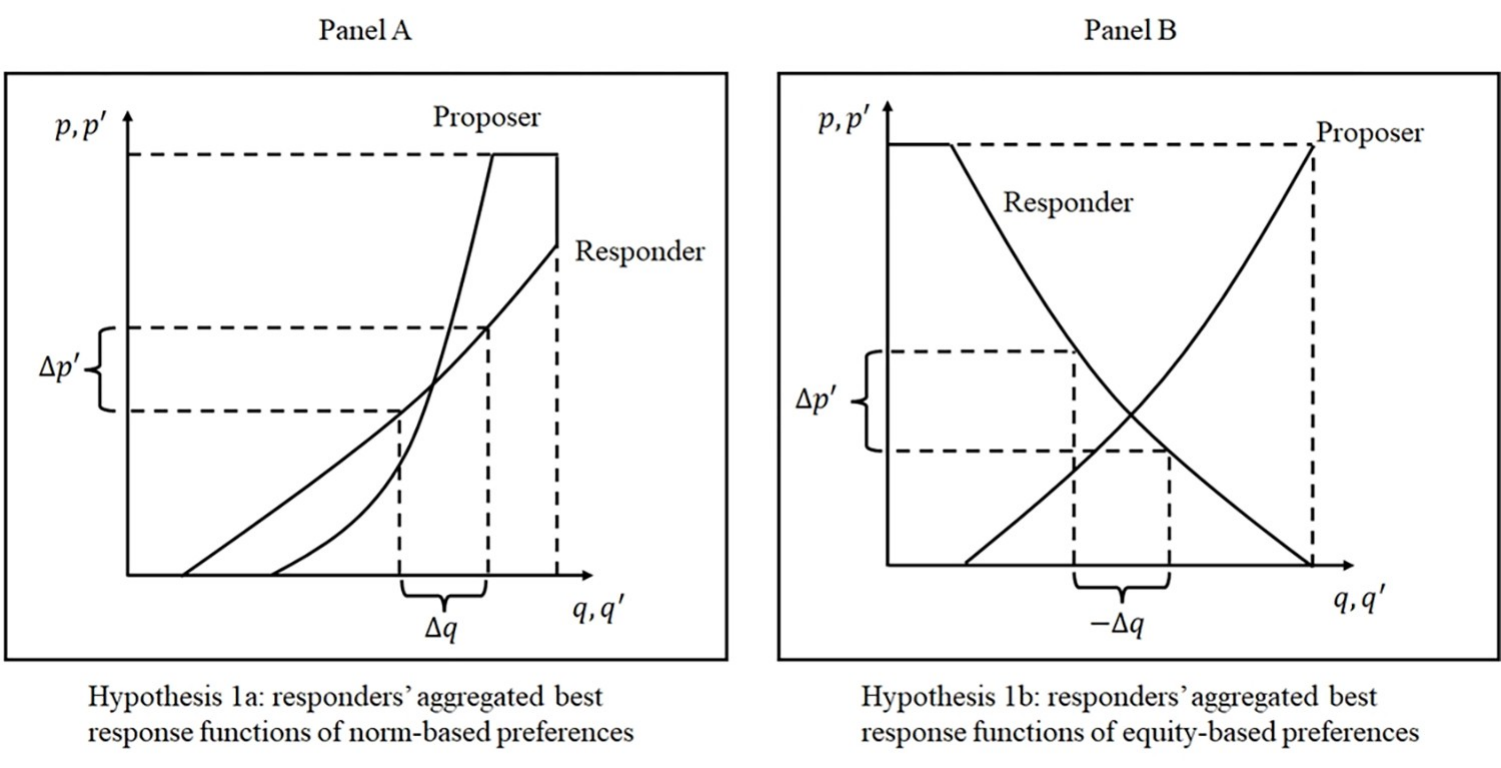
Illustration of hypotheses.

The difference in *p*′ between the neutral and hierarchical treatments, denoted as Δ*p*′, represents the influence of framing on beliefs. This difference is visualized as a shift in *p*′ along the aggregated best response curve in Fig 2. Hence, framing is the key independent variable in our analysis, while the dependent variable is either *q* or *p*. The belief *p*′ serves as the mediator through which framing impacts decisions. The sign of Δ*q*/Δ*p*′ serves to confirm which hypothesis is supported. In retrospect, we observe that the magnitude of Δ*p*′ is significant only among the subgroup of student cadres, suggesting that the cadre status moderates the treatment effect on *p*′.

### Experiment design

The experiment was approved by the research ethics committee of our research institute (CCBEF). We conducted it according to the ethical guidelines of the Declaration of Helsinki. All participants’ signed consent forms were obtained before the experiment, which began on May 28^th^, 2022, and ended the same day. Experimental instructions, surveys and a blank, sample copy of the subject consent form can be found in the S1 Appendix.

The design of the experiment replicates the embezzlement game. In order to enhance the unethical nature of embezzlement, the endowment that the players are about to split comes from a project’s return, where both the proposer and responder have 50% of its share. In addition, they were mandated to invest all of them into the project, but only one can propose how to distribute the return. The experiment is a one-shot game conducted with paper and pencil to eliminate the potential confounds of reputation or learning effect.

Upon arrival at the laboratory, participants in the baseline group were randomly allocated to either Group A (proposers) or Group B (responders). They were seated in separate rooms to ensure no subsequent interaction between the two groups. Experimenters provided oral instructions for the tasks and were available to address any questions privately. Participants were required to demonstrate their understanding of the procedures by correctly answering quiz questions. The payment structure was designed to be one-sided and blinded, ensuring participants were unaware of the other subjects’ earnings. A strict non-communication rule was enforced to prevent any form of collusion or coordination during the experiment. The strategy method was employed to efficiently capture decisions contingent on various possible scenarios.

The experiment procedure consists of three parts. The first part is the embezzlement game, in which subjects were randomly paired anonymously. Each received an initial 50-token endowment (1 token = 1 RMB Chinese Yuan) to jointly invest in a project with a 1/8 chance of an 80% loss; otherwise, no loss. With a 20-token outcome, each party received 10 tokens. But with 100 tokens, the Proposer chose between:

- Proposal 1: If the final value of the project is 100, I will receive 90 tokens, my partner will receive 10 tokens;
- Proposal 2: If the project’s final value is 100, I will receive 50 tokens, my partner will receive 50 tokens.

The responder does not know the final project value, and the responder needs to choose for the following two scenarios:

- I “Accept” or I “Reject” when 50 tokens are offered;
- I “Accept” or I “Reject” when 10 tokens are offered.

If accepted, payoffs were determined by nature or choice. If rejected, both received 0 tokens. A dice roll privately determined the project value unseen by Responders.

The second phase of the experiment consisted of a survey designed to ascertain participants’ beliefs, serving as proxies for descriptive norms. Proposers were asked to estimate the general acceptance rate among responders when presented with an offer of 10 tokens, while responders were tasked with estimating the percentage of proposers who elected Proposal 1. Additionally, participants were asked to predict their counterparts’ responses to these questions. To prevent any influence on the decision-making process or inducing a “think harder effect”, belief elicitation was conducted post-decision. To discourage participants from reporting arbitrary probabilities, a reward system was implemented whereby accurate belief estimation that closely aligns with actual population frequencies will be rewarded with three tokens per question—this incentive aimed to motivate participants to provide well-considered estimations.

The third part of the experiment consisted of a psychological questionnaire, Test of Self-Conscious Affect–3 (TOSCA-3 [35]) and demographic survey. TOSCA-3 is mainly used to measure the subjects’ level of sensitivity to guilt and shame.

In the hierarchical treatment, proposers were referred to as “Guan” (officials) and responders as “Min” (commoners). Other than that, the experiment procedure was identical to that of the baseline treatment.

A total of 264 students participated in the experiment, with an equal number of 132 students assigned to each baseline and hierarchical treatment. Within each group, there were 66 proposers and 66 responders. All participants were undergraduate students from a university in Chengdu, China.

## Results

Table 1 presents the demographic statistics. The first two columns provide the mean values or proportions of demographic variables for subjects in both the baseline and hierarchical treatments. These variables were compared between the two treatments using Wilcoxon-Mann-Whitney (W-M-W) rank-sum tests, with the associated p-values listed in the third column. The results reveal no significant differences between the demographic profiles of the treatment groups.

**Table 1:** Demographic statistics of the two treatments.

Demographic variables	Baseline (N = 132)	Hierarchical (N = 132)	Baseline VS. Hierarchical	Ordinary VS. Cadres
Student Cadre			0.615	
Yes	37.88%	40.91%		
No	62.12%	59.09%		
Gender			0.805	0.663
Male	46.21%	44.70%		
Female	53.79%	55.30%		
Age	18.73 (1.15)	18.76 (1.08)	0.686	0.069*
Race			0.556	0.330
Han	90.15%	87.88%		
Non-Han	9.85%	12.12%		
Expenditure			0.681	0.252
Less than 500 yuan	19.70%	27.27%		
500–1000 yuan	65.15%	53.03%		
1000–1500 yuan	14.39%	17.42%		
More than 1500 yuan	0.76%	2.27%		
Grade			0.710	0.630
Freshman	38.64%	38.64%		
Sophomore	57.58%	53.79%		
Junior	3.79%	7.58%		
Major			0.492	0.727
Economics and Business	25.76%	29.55%		
Others	74.24%	70.45%		
GPA-Rank			0.676	0.680
At the Top	3.79%	8.33%		
Above the Average	40.91%	37.12%		
On the Average	49.24%	38.64%		
Below the Average	5.30%	13.64%		
At the Bottom	0.76%	2.27%		
Guilt	64.47 (6.48)	64.64 (5.50)	0.985	0.288

Note: Standard errors are given in brackets. Guilt measures the subject’s degree of guilt aversion (from TOSCA-3); p-values of the W-M-W rank-sum test are given in columns 3 and 4; * indicates significant at 10% level.

The fourth column of Table 1 contrasts ordinary students with student cadres, motivated by a critical experimental finding that student cadres were influenced differently by hierarchical framing compared to their peers. In the Chinese context, the term “student cadre” bears connotations of a “student official”, as these individuals are often connected with officially recognized University organizations or departments and may be more accustomed to bureaucratic culture than other students. The voluntary recruitment for student cadres suggests that individuals who are more amenable to hierarchical norms might self-select into these roles. Other factors may influence the treatment effect related to student cadre status, such as academic achievement, motivation level, or career aspirations. Further investigation is needed to fully understand the influence of cadre status on the observed treatment effect. However, the random assignment of student cadres to treatments, like other students, ensures that the validity of the treatment effect is maintained. In the fourth column of Table 1, the outcomes of W-M-W tests between student cadres and other students are reported. Except for student cadres being slightly older, there are no significant differences in gender, ethnicity, wealth, academic performance, or guilt sensitivity.

As anticipated by the theoretical framework, every recipient who was offered 50 tokens chose to accept. Consequently, our analysis will predominantly concentrate on the responders’ decisions when faced with a 10-token offer. A necessary question to address before testing Hypothesis 1 is whether the framing effect induces variability in responders’ beliefs about embezzlement. Table 2 encapsulates the responders’ first-order beliefs regarding embezzlement across different groups.

**Table 2:** Responder’s first-order beliefs.

**Panel A: Responder’s first-order beliefs**			
	**All Students**	**Ordinary Students**	**Student Cadres**
**Basement**	0.512 (0.319)	0.498 (0.325)	0.535 (0.314)
**Hierarchical**	0.595 (0.315)	0.450 (0.282)	0.793 (0.245)
**Basement VS. Hierarchical**	0.067* /0.197	0.753 /0.399	0.001*** /0.003***
**Panel B: Responder’s first-order beliefs segregated by decisions**			
	**Accept**	**Reject**	**Accept VS. Reject**
**Basement**	0.598 (0.314)	0.341 (0.258)	0.001*** /0.004***
**Hierarchical**	0.683 (0.283)	0.379 (0.290)	0.000*** /0.000***

Note: Standard errors are presented in parentheses The *p*-values of one-tailed t test and W-M-W rank-sum test are given in the cells that compare the two groups; *, **, and *** indicates significant at 10%, 5%, and 1% level respectively.

In Panel A of Table 2, the hierarchical framing appears to increase the overall beliefs about embezzlement across the full sample, but this increase does not reach a high significance level. However, within the student cadre subgroup, we observe a substantial belief shift. Hierarchical framing notably elevates first-order beliefs (*p*′) among student cadres compared to the baseline treatment, as evidenced by significant results in both the t-test (p<0.001) and the W-M-W test (p = 0.003). On the other hand, we do not observe a similar effect for the other students.

Panel B of Table 2 categorizes the full sample based on their reaction to the low offer—accepting or rejecting it. Beliefs about embezzlement among those who rejected the offer are significantly lower than those who accepted it in both treatment conditions. This finding aligns with the norm-based preference model, posing that responders with a lower perceived norm are more inclined to accept an unfavorable offer. Nevertheless, the relationship between beliefs and behavior could be confounded by extraneous variables, so our analysis will concentrate on the treatment effect, as detailed in Table 3.

**Table 3:** Responder’s decisions.

	Baseline		Hierarchical		Baseline VS. Hierarchical
	Accept	Reject	Accept	Reject	
**All Students**	66.67%	33.33%	71.21%	28.79%	0.573/0.354
**Ordinary Students**	72.50%	27.50%	55.26%	44.74%	0.113/0.088*
**Student Cadres**	57.69%	42.31%	92.86%	7.14%	0.003***/0.003***
**Ordinary students VS. Cadres**	0.212/0.164		0.000***/0.000***		

Note: The p-values of Chi-square test and Fisher’s exact test are given in the last column to compare the two groups.

Table 3 summarizes the effect of framing on responders’ decisions. Utilizing the Chi-square test (p = 0.573) and Fisher’s exact test (p = 0.354), no significant variance in acceptance rates between treatments was found for the full sample. Within specific subgroups, both ordinary students (29 of 40; 72.50%) and student cadres (15 of 26; 57.69%) exhibited comparable decision patterns in the baseline treatment, with no significant difference detected (Chi-square p = 0.212, Fisher’s p = 0.164). However, in the hierarchical treatment, student cadres (26 of 28; 92.86%) demonstrated a considerably higher propensity to accept low offers compared to ordinary students (21 of 38; 55.26%), as indicated by one-sided Chi-square test (p<0.001) and Fisher’s test (p<0.001). Specifically, student cadres were markedly more inclined to accept low offers under hierarchical framing (Chi-square p = 0.003, Fisher’s p = 0.003), while ordinary students showed a marginal decrease in their acceptance rates (Fisher’s p = 0.088).

Combining the findings from Tables 2 and 3, the results are consistent with Hypothesis 1a: that hierarchical framing, when inducing sufficient variations in the descriptive norm of corruption, leads to a positive association between responders’ beliefs about embezzlement and their acceptance of such acts. To account for other factors that might concurrently affect the outcome, we employ the Probit regression for a more comprehensive investigation.

In Table 4, Model 1 establishes the baseline regression. Model 2 adds an interaction term to assess the influence of cadre status as a moderating variable. Models 1, 3, and 5 employ the causal step approach defined by Baron and Kenny (1986) to test the mediating effect of *p*′ on the treatment effect. Specifically, Model 3 examines whether the framing induces variability in the mediator, while Model 5 investigates the impact of mediating variables on decision-making, as indicated by the coefficient of *p*′. Similarly, models 2, 4, and 6 test whether the cadre status moderates the mediating effect.

**Table 4:** The regressions on responder’s decisions and beliefs.

Dependent Variable	Accept the low offer		*p*′		Accept the low offer	
	(1)	(2)	(3)	(4)	(5)	(6)
**Framing**	0.137	-0.492	0.046	-0.070	0.012	-0.463
	(0.255)	(0.340)	(0.058)	(0.071)	(0.272)	(0.367)
**Student Cadre**		-0.349		0.061		-0.512
		(0.340)		(0.080)		(0.340)
Framing* Cadre		1.826***		0.267**		1.632***
		(0.562)		(0.104)		(0.558)
***p*′**					2.174***	1.951***
					(0.457)	(0.498)
**Guilt**	-0.046**	-0.048*	-0.004	-0.005	-0.048*	-0.045*
	(0.023)	(0.025)	(0.005)	(0.004)	(0.025)	(0.026)
**Gender**	-0.016	-0.049	0.105*	0.089*	-0.273	-0.262
	(0.244)	(0.255)	(0.055)	(0.051)	(0.261)	(0.269)
**Age**	-0.073	-0.079	-0.019	-0.015	-0.062	-0.052
	(0.146)	(0.155)	(0.037)	(0.035)	(0.156)	(0.163)
**Race**	0.137	0.193	-0.117	-0.113*	0.454	0.499
	(0.362)	(0.362)	(0.076)	(0.067)	(0.385)	(0.395)
**Expenditure Dummy**	-0.026	0.213	-0.012	0.038	-0.043	0.141
	(0.354)	(0.374)	(0.069)	(0.065)	(0.385)	(0.418)
**Major Dummy**	0.202	0.013	0.186***	0.137**	-0.221	-0.367
	(0.313)	(0.331)	(0.056)	(0.053)	(0.338)	(0.358)
**Grade**						
Sophomore	0.347	0.436	0.103	0.115	0.128	0.169
	(0.327)	(0.350)	(0.079)	(0.073)	(0.347)	(0.367)
Junior	-0.269	-0.036	0.189	0.234	-0.734	-0.577
	(0.704)	(0.749)	(0.165)	(0.147)	(0.694)	(0.752)
**Score**						
= Average	-0.207	-0.066	-0.074	-0.024	-0.135	-0.173
	(0.420)	(0.484)	(0.098)	(0.085)	(0.423)	(0.510)
> Average	0.066	0.223	-0.054	-0.001	0.169	0.187
	(0.433)	(0.499)	(0.094)	(0.079)	(0.444)	(0.526)
**R2**	0.065	0.154	0.143	0.267	0.210	0.256
**Observations**	132	132	132	132	132	132

Note: Robust standard errors are reported in parentheses; *p*′ represents the responder’s first-order belief about embezzlement; framing = 0 indicates baseline treatment; guilt refers to the TOSCA-3 measurement of guilt aversion; gender = 0 means female and 1 means male; Race = 1 is referred to as Han ethnicity and 0 as otherwise; Expenditure Dummy = 0 indicates spending less than 1000 yuan and 1 means otherwise; Major Dummy = 0 denotes major in Economics or Business.

The findings from Models 1 and 2 indicate a substantial treatment effect among the subgroup of student cadres but not within the non-cadre student population. The framing coefficient in Model 3 suggests that hierarchical framing may not produce sufficient variation in beliefs (descriptive norm) across the entire sample. However, the significant coefficient of the interaction term in Table 4 reveals that framing significantly alters the beliefs of student cadres. In both Model 5 and Model 6, the influence of the mediator *p*′ on decision outcomes remains significant, aligning with the trends observed in Tables 2 and 3. In summary, the data lead to the following conclusion:

**Result 1**. Hierarchical framing significantly elevates student cadres’ beliefs about embezzlement and their likelihood of accepting low offers, but this effect does not extend to other students.

On the proposer’s side, the interest lies in whether proposers alter their decisions based on their beliefs about responders’ reactions under hierarchical framing. Table 5 presents the average beliefs denoted as *q*′ between the baseline and hierarchical treatments. The results suggest that student cadres exhibit higher beliefs about responders accepting the low offer, with p-values from the one-tailed t-test and W-M-W test at 0.022 and 0.082, respectively. However, these effects do not reach statistical significance for ordinary students.

**Table 5:** Proposer’s first-order beliefs.

**Panel A: Proposer’s first-order beliefs**			
	**All Students**	**Ordinary Students**	**Student Cadres**
**Baseline**	0.598 (0.346)	0.655 (0.347)	0.500 (0.327)
**Hierarchical**	0.682 (0.287)	0.683 (0.290)	0.681 (0.288)
**Baseline VS. Hierarchical**	0.067* /0.273	0.348 /0.895	0.022** /0.082*
**Panel B: Proposer’s first-order beliefs segregated by different decisions**			
	**Proposal 1**	**Proposal 2**	**Proposal 1 VS. Proposal 2**
**Baseline**	0.770 (0.287)	0.456 (0.328)	0.000*** /0.000***
**Hierarchical**	0.784 (0.222)	0.462 (0.292)	0.000*** /0.000***

Note: Standard errors are presented in parentheses; the p-values of the one-tailed t-test and W-M-W rank-sum test are given in the cells that compare the two groups.

Panel B of Table 5 suggests that proposers with differing choices hold distinct beliefs about the responders’ behavior (p < 0.001 for both tests). These findings align with the hypothesis that proposers anticipating responders’ compliance are more prone to embezzlement.

Table 6 examines the extent of embezzlement across different treatment conditions. In the baseline treatment, 30 out of 66 proposers (45.45%) opted for proposal 1, compared to 45 out of 66 proposers (68.18%) who chose the same in the hierarchical treatment. Statistical analysis using the Chi-square test (p = 0.008) and one-sided Fisher’s exact test (p = 0.007) leads to rejecting the null hypothesis, asserting no difference between the treatments. This outcome indicates that the hierarchical framing significantly influences proposers towards more corrupt behavior.

**Table 6:** Proposer’s decisions.

	Baseline		Hierarchical		Baseline VS. Hierarchical
	Proposal 1	Proposal 2	Proposal 1	Proposal 2	
**All Students**	45.45%	54.55%	68.18%	31.82%	0.008***/0.007***
**Ordinary Students**	50.00%	50.00%	57.50%	42.50%	0.496/0.323
**Student Cadres**	37.50%	62.50%	84.62%	15.38%	0.001***/0.001***
**Ordinariness VS. Cadres**	0.327/0.235		0.021**/0.019**		

Note: p-values of the one-sided Chi-square test and Fisher exact test are given for comparison.

Mirroring the pattern observed on the responder side, the influence on proposer behavior is similarly attributed to student cadres. Half of the ordinary students (21 out of 42) and 37.5% of the student cadres (9 out of 24) chose proposal 1 in the baseline treatment. Neither the one-sided Chi-square test nor Fisher’s exact test revealed a significant discrepancy in the levels of corrupt behavior between the two subgroups (p = 0.327, p = 0.235). Contrastingly, under hierarchical framing, the choices of student cadres diverged markedly. Of the 66 individuals, a significant majority of student cadres (22 out of 26 or 84.6%) chose proposal 1 as opposed to 57.5% of ordinary students (23 out of 40). These statistical tests invalidate the null hypothesis of equal propensity for corruption across subgroups (Chi-square p = 0.021, Fisher’s p = 0.019).

Shifting to a parametric approach, Table 7 presents regression outcomes on embezzlement decisions, employing methodologies analogous to those used in Table 4. The results from Models 1 and 2 confirm that both the treatment effect on decisions and the moderating role of cadre status are significantly positive. This suggests increased embezzlement under hierarchical framing, primarily driven by student cadres. Model 3 considers the impact of framing on the mediator *q*′ while Model 4 examines whether the influence is moderated by the cadre status of the student. The findings from both models are not statistically significant, suggesting that hierarchical framing may not sufficiently alter proposers’ beliefs.

**Table 7:** The regressions on the proposer’s decisions and beliefs.

	Choosing Proposal 1		*q*′		Choosing Proposal 1	
	(1)	(2)	(3)	(4)	(5)	(6)
**Framing**	0.519**	0.094	0.060	0.021	0.485*	0.068
	(0.241)	(0.302)	(0.056)	(0.073)	(0.266)	(0.332)
**Student Cadre**		-0.264		-0.147*		0.100
		(0.347)		(0.087)		(0.415)
Framing * Cadre		1.236**		0.116		1.306**
		(0.504)		(0.116)		(0.573)
***q*′**					2.280***	2.517***
					(0.457)	(0.498)
**Guilt**	-0.017	-0.022	0.008	0.008	-0.036	-0.043*
	(0.020)	(0.021)	(0.005)	(0.005)	(0.024)	(0.026)
**Gender**	0.090	0.043	0.044	0.036	-0.041	-0.118
	(0.259)	(0.271)	(0.062)	(0.062)	(0.323)	(0.335)
**Age**	0.120	0.194	0.018	0.014	0.134	0.261*
	(0.145)	(0.148)	(0.037)	(0.036)	(0.141)	(0.153)
**Race**	0.200	0.159	0.049	0.053	0.088	0.010
	(0.531)	(0.522)	(0.138)	(0.141)	(0.570)	(0.596)
**Expenditure Dummy**	0.669**	0.629*	0.100	0.093	0.583*	0.540
	(0.317)	(0.328)	(0.070)	(0.073)	(0.318)	(0.329)
**Major Dummy**	0.701***	0.720***	0.062	0.059	0.687**	0.753**
	(0.260)	(0.261)	(0.060)	(0.061)	(0.283)	(0.296)
**Grade**						
Sophomore	-0.235	-0.364	-0.009	0.003	-0.292	-0.488
	(0.308)	(0.314)	(0.074)	(0.071)	(0.353)	(0.363)
Junior	0.042	-0.036	-0.086	-0.072	0.138	-0.001
	(0.564)	(0.552)	(0.124)	(0.116)	(0.568)	(0.575)
**Score**						
= Average	-0.172	-0.175	-0.053	-0.047	-0.151	-0.156
	(0.426)	(0.455)	(0.099)	(0.098)	(0.444)	(0.450)
> Average	0.120	0.063	0.010	0.020	0.062	-0.011
	(0.414)	(0.444)	(0.094)	(0.094)	(0.401)	(0.401)
**R2**	0.129	0.169	0.077	0.102	0.303	0.360
**Observations**	132	132	132	132	132	132

Models 5 and 6 explore the influence of the mediating variable on embezzlement decisions. The coefficients for *q*′ indicate that a proposer’s belief in the responder’s willingness to accept a low offer correlates positively with the likelihood of choosing to embezzle. Nonetheless, this tendency may not directly result from the experimental manipulation since the proposers’ beliefs about responders do not significantly differ between treatments. The results suggest that the proposers’ marked response to the framing is likely not due to their expectations of responder compliance but rather due to their beliefs about the prevalence of embezzlement among other proposers. Combing results of both parametric and non-parametric analysis, we derive the following:

**Result 2**. Hierarchical framing significantly increases the likelihood of embezzlement across the entire student cohort, yet it only marginally increases student cadres’ beliefs about the responders’ acceptance rates for the low offer.

Summarizing the previous analysis, we can conclude that result 1 robustly supports hypothesis 1a. Despite the hierarchical framing not inducing substantial variation in beliefs as expected, there is a notable increase in the acceptance rate for the low offer among those samples whose beliefs were influenced. This suggests that the impact of the descriptive norm on victims’ compliance may be more pronounced than the experimental results indicate, given that individuals’ perceived norms are likely more affected by real-life experiences than by the framings in a lab environment.

On the proposer’s side, Result 2 only offers partial evidence for hypothesis 2, mainly because the hierarchical framing does not significantly alter proposers’ beliefs. Nevertheless, the correlation between beliefs and decisions remains robust across treatments. This finding implies that while there is no clear indication that proposers adjust their expectations regarding changes in compliance due to hierarchical norms, they are more inclined to engage in embezzlement when they anticipate a lower likelihood of punishment from responders.

## Discussion

Our experiment lends support to the hypothesis that a corruptive social norm facilitates corruption by promoting victims’ compliance. When victims perceive corruption as widespread and expect outcomes to be inequitable, they become less inclined to penalize corrupt behavior. This finding supplements the existing literature on descriptive norms and corruption, enhancing our understanding of why corruption often evolves into a self-perpetuating cycle. Beyond the primary outcomes, our study also reveals that framing significantly influences proposers’ decisions but not responders’ decisions. This suggests that those who initiate corruption are more susceptible to hierarchical framing than the victims, likely because acting on hierarchical cues aligns with the self-interest of the corrupt, whereas it is detrimental to the victims’ interests. Consequently, bureaucratic culture might more severely undermine the descriptive norms of government officials than civilians. Therefore, the potential detrimental influence of hierarchical framings on descriptive norms should be carefully considered before they are implemented in government institutions.

Existing research on descriptive norms and corruption has established that perceptions of corrupt behavior positively correlate with decisions to engage in corruption. The belief that “everyone else is doing it” forms a significant motivation for corrupt actions [1,2,18,19]. In addition to internal motivations, corruption is also influenced by external constraints, such as social judgment and punishment [23,25–27]. This paper contributes to the literature by examining how descriptive norms affect these external constraints, specifically through the victims’ decision to punish possible embezzlement. Our findings, combined with those from previous studies, support the notion that corruption tends to evolve into a self-reinforcing cycle. More pervasive corruption not only provides stronger incentives for individuals to engage in corrupt behavior but also diminishes the likelihood of punitive actions being taken against them.

A potential limitation of our experimental approach is that the efficacy of the treatment is contingent upon cultural context. The propensity of individuals to respond to hierarchical framing is deeply entwined with a history of centralized governance and entrenched hierarchical norms. Consequently, the replicability of the framing effects documented in this study might be limited by cultural factors. Nevertheless, it is essential to recognize that the primary objective of using framing effects is to engender exogenous shifts in beliefs and decisions. Therefore, alternative techniques that elicit comparable disruptions in descriptive norms could be employed to test the connection between norms and compliances.

Lastly, the mechanisms elucidated within the norm-based fairness model may have broader applicability. From a more general perspective, the association between corruptive norms and compliance can be interpreted as a consequence of players’ other-regarding preferences skewed by social norms’ degradation. This mechanism could extend to a variety of contexts. For example, a well-established observation in public goods games is the decline in contributions over time. From the perspective of social norms, this gradual reduction could be attributed to the deterioration of descriptive norms.

## Supporting information

S1 Appendix Experimental instructions and surveys. (DOCX)

S1 File Data of experiment. (ZIP)
