# Supplementary material for: Tolerance for corruption and descriptive social norm: An experimental study of embezzlement
Source: PLoS One. 2024 May 20;19(5):e0303558. doi: 10.1371/journal.pone.0303558 (PMC11104634; doi:10.1371/journal.pone.0303558)
Supplement: S1 Appendix — (DOCX) [file pone.0303558.s001.docx]

**Tolerance for Corruption and Descriptive Social Norm: An Experimental Study of Embezzlement By Sen Tian and Liangfo Zhao**

**Appendix**

This appendix includes the experimental instructions. The experiment is divided into two stages:

The game: we provide the same game instruction and different decision sheets for the two players: proposers and responders.

The survey: this survey collect psychological and demographic information from the subjects, and they are the same for both the proposers and responders.

The instructions in the following sections have been translated from Chinese to English, and they are presented in the same chronological order as the subjects received in the experiment.

Agreement to participation in social and behavioral research

I consent to participate in this experiment.

I have already been clearly informed by the chief organizer Sen Tian (Assistant Professor of Southwestern University of Finance and Economics) and his deputy about the experiment procedure. I have clearly understood how payment is distributed and how long does experiment take. I also know my personal information will be kept confidential, and all the information I received is free of deception.

I now admit I can get more information from the experimenter if I have any question about the experiment.

Moreover, I know that I can quit the experiment at any time I want, without causing me any harm.

If I had any problem concerning my right and interests as a participant of the experiment, I can contact the host through mail [tiansen@swufe.edu.cn](mailto:tiansen@swufe.edu.cn) or telephone 02887098046.

Finally, I will sign to admit I have fully understood the content above.

Participant:

Host of the experiment

(or its deputy):

Date

Email address

Contact

**Instruction (for all subjects)**

Welcome to our lab experiment. You are about to make some decisions on which your experimental income may depends. Your payoff may also be influenced by luck and other players’ decisions. We will also pay you 5 tokens as our gratitude for your participation. The exchange rate of token to RMB is 1:1. The information about your income will be kept confidential and you will receive the payment at the end of experiment in cash. Please read this manual carefully, it will guide you through all the stages of this experiment.

You are not supposed to be intellectually tested when answering the questions in the experiment, and there are no wrong answers. Please make your choice based on your preferences in real world.

This experiment is consists of three stages, each includes several different tasks. Before entering the lab, we will ask you to randomly draw a number card from the card pool. The number you draw will be your unique identification number throughout the experiment, please keep it alongside until you receive the payment. There are totally 132 cards, starting from A101 to A166 for A, and B101 to B166 for B. Our staff will guide you to your sit according to your ID number. At the end of the sessions, we will only keep track of your ID number in the experiment and your decisions for our research purposes.

Finally, please keep your phone switched off and do not communicate with each other. If you violated any of them, we will have to ask you to leave without any payoff. Please keep the instruction manual tidy. If you need to take notes, please write on draft papers. Fill your answer on the experimental cards. We will retrieve instruction manual, experimental cards, draft papers and pencil. If you have any questions during the experiment, please raise your hand and our staff will approach you.

Now, please draw your identification number. Then, subjects who have number between A101 and A166 go to classroom 618, and subjects who have number between B101 and B 166 go to classroom 620. Please take the seat where your number is marked.

**Experimental Procedures (for all subjects)**

This experiment is consists of two stages.

Stage 1, dual decision making game. Subjects whose ID number is between A101 and A166 are called subjects “A”, and subjects whose ID number is between B101 and B166 are called subjects “B”. Computer will randomly match one member of A to a member of B, so that two of you consists a pair. Your personal information (including ID number) will remain confidential during the whole experiment.

At the beginning of this stage, you and your partner will each get 50 tokens as endowment and also have to invest all your endowment into a project, the value of this project are equally earned by you and your partner. The value of project have 1/8 chance to shrink into 20 tokens, and 7/8 chance to remain 100 tokens. Whether the value shrinks depends on rolling a 8-sided dice. If it turned out to be 1, value will go down to 20, if not, the value remains to be 100. But Only A can see the result of the roll of dice, and only A can decide how to distribute the value of project.

Before rolling the dice, A will be asked to choose one from the following two proposals to decide how the value of project is divided if the result of dice is not 1:

**Proposal 1:** if the value of project is 100, A get 90 and B get 10;

**Proposal 2:** if the value of project is 100, A get 50 and B get 50.

Before rolling the dice, B will be also asked to make the decisions under following scenario:

**When receiving 10**, you choose to: Accept

Reject

**When receiving 50**, you choose to: Accept

Reject

The decision made by you and your partner will be kept secrecy during the whole experiment. When both of you have made your choices, our staff will provide dices and determine your experimental payoff according to the following rules:

If the dice turned out to be 1, the value of project will be 20. If B chose agree when receiving 10, both A and B will get 10 as payoff. Otherwise, both of you get 0.

If the dice turned out to be a number between 2 and 8, the value of project will be 100. If A chose proposal 1 and B chose agree when receiving 10, both A will get 90 and B will get 10 as payoff; if B chose reject when receiving 10, both of A and B get 0. If A chose proposal 2 and B chose agree when receiving 50, both A and B will get 50 as payoff; if B chose reject when receiving 50, both of A and B get 0.

Or in another way, the payoff rules can be summarized as :

If the die lands on 1

| B’s choice | A’s payoff | B’s payoff |
| --- | --- | --- |
| Agree when receiving 10 | 10 | 10 |
| Reject when receiving 10 | 0 | 0 |

If the die lands on 2-8

| A’s choice | B’s choice | A’s payoff | B’s payoff |
| --- | --- | --- | --- |
| Proposal 1 | Agree when receiving 10 | 90 | 10 |
|  | Reject when receiving 10 | 0 | 0 |
| Proposal 2 | Agree when receiving 50 | 50 | 50 |
|  | Reject when receiving 50 | 0 | 0 |

Please be aware that when B receive 10, he or she cannot tell whether it is because the die lands on 1 or A chose proposal 1.

Before the game starts, we will ask you to finish a quiz to make sure that you understand the rules. After which you can make your decisions, and then we will send the staff to bring the dice to you.

Stage 2 is the psychological survey and demographic survey.

**Quiz (for all subjects)**

Your ID number is______

This quiz is designed to test whether you have understood the game rules in the previous instruction. There is only 1 **correct** answer to each question. You need to give the right answer to every question before you can proceed to the next phase.

1. if player A chose “proposal 1”, and the matching player B chose “agree” when receiving 10 tokens, and the dice landed on 2-8, please calculate the payoff of player A and B:

A. 0 ,0 ； B. 0 ,100 ； C. 50 ,50 ； D. 90 ,10 ； E. 10,10

2. if player A chose “proposal 1”, and the matching player B chose “reject” when receiving 10 tokens, and the dice landed on 2-8, please calculate the payoff of player A and B:

A. 0 ,0 ； B. 0 ,100 ； C. 50 ,50 ； D. 90 ,10 ； E. 10,10

3. if player A chose “proposal 1”, and the matching player B chose “agree” when receiving 10 tokens, and the dice landed on 1, please calculate the payoff of player A and B:

A. 0 ,0 ； B. 0 ,100 ； C. 50 ,50 ； D. 90 ,10 ； E. 10,10

4. if player A chose “proposal 1”, and the matching player B chose “reject” when receiving 10 tokens, and the dice landed on 1, please calculate the payoff of player A and B:

A. 0 ,0 ； B. 0 ,100 ； C. 50 ,50 ； D. 90 ,10 ； E. 10,10

5. if player A chose “proposal 2”, and the matching player B chose “agree” when receiving 50 tokens, and the dice landed on 2-8, please calculate the payoff of player A and B:

A. 0 ,0 ； B. 0 ,100 ； C. 50 ,50 ； D. 90 ,10 ； E. 10,10

6. if player A chose “proposal 2”, and the matching player B chose “agree” when receiving 50 tokens, and the dice landed on 1, please calculate the payoff of player A and B:

A. 0 ,0 ； B. 0 ,100 ； C. 50 ,50 ； D. 90 ,10 ； E. 10,10

7. if player A chose “proposal 2”, and the matching player B chose “reject” when receiving 50 tokens, and the dice landed on 2-8, please calculate the payoff of player A and B:

A. 0 ,0 ； B. 0 ,100 ； C. 50 ,50 ； D. 90 ,10 ； E. 10,10

8. if player A chose “proposal 2”, and the matching player B chose “reject” when receiving 50 tokens, and the dice landed on 1, please calculate the payoff of player A and B:

A. 0 ,0 ； B. 0 ,100 ； C. 50 ,50 ； D. 90 ,10 ； E. 10,10

|  | 1 | 2 | 3 | 4 | 5 | 6 | 7 | 8 |
| --- | --- | --- | --- | --- | --- | --- | --- | --- |
| Your answer |  |  |  |  |  |  |  |  |

**Experimental Card 1 for Proposers**

Your ID number is______

According to the experimental rules on instruction manual, please deicide your action is ______ when the die lands on 2-8 (A, proposal 1; B proposal 2)

Proposal 1: if the value of project is 100 tokens, A receives 90 tokens, B receives 10 tokens.

Proposal 2: if the value of project is 100 tokens, A receives 50 tokens, B receives 50 tokens.

**Experimental Card 2 for Proposers**

Your ID number is______

Survey 1

There are no wrong answers in this survey, please read the questions carefully, and make your decision based on your real life experience. Additionally, we offer extra bonus if your answer is close to the outcome. We will pay the bonus in cash along with your experimental payoff at the end of this experiment.

1. Please estimate the overall acceptance ratio of all subjects B when they receive 10 tokens. If your guess falls close to the real ratio, you will be awarded with 3 tokens.

1： 0%-10% 2：10%-20% 3：20%-30% 4：30%-40% 5：40%-50%

6： 50%-60% 7：60%-70% 8：70%-80% 9：80%-90% 10：90%-100%

1. We asked the player B whom you matched with to estimate the probability that you will choose proposal 1, please guess which of the following interval his answer may fall in. If your guess is close to the real ratio, you will be awarded with 3 tokens.

1： 0%-10% 2：10%-20% 3：20%-30% 4：30%-40% 5：40%-50%

6： 50%-60% 7：60%-70% 8：70%-80% 9：80%-90% 10：90%-100%

Now our experimental staff will approach you with a 8 sided die, please roll the dice and write the result on this page. Noted that the subject B you are playing with cannot see this result. Your die landed on _____.

**Experimental Card 1 for Responders**

Your ID number is______

According to the experimental rules on instruction manual, please deicide:

1, when you receive 10 tokens, you choose to ____ the offer.

A, Accept ; B, Reject

2, when you receive 50 tokens, you choose to ____ the offer.

A, Accept ; B, Reject

**Experimental Card 2 for Responders**

Your ID number is______

Survey 1

There are no wrong answers in this survey, please read the questions carefully, and make your decision based on your real life experience. Additionally, we offer extra bonus if your answer is close to the outcome. We will pay the bonus in cash along with your experimental payoff at the end of this experiment.

3. Please estimate of all subjects A, how many of them chose proposal 1 (A takes 90 tokens, B receives 10 tokens). If your guess falls close to the real ratio, you will be awarded with 3 tokens.

1： 0%-10% 2：10%-20% 3：20%-30% 4：30%-40% 5：40%-50%

6： 50%-60% 7：60%-70% 8：70%-80% 9：80%-90% 10：90%-100%

4. We asked the player A whom you matched with to estimate the probability that you will accept the 10 tokens offer, please guess which of the following interval his answer may fall in. If your guess is close to the real ratio, you will be awarded with 3 tokens.

1： 0%-10% 2：10%-20% 3：20%-30% 4：30%-40% 5：40%-50%

6： 50%-60% 7：60%-70% 8：70%-80% 9：80%-90% 10：90%-100%

**Survey 1 for All Subjects (Demographic Survey)**

Your ID number is______

The data of the following 2 surveys will be kept confidential.

1. You are _____(male or female)

2. You are from _____ province

3. Your birth date is ____________

4. Your nationality is ____________

5. Your major is __________

(1) Business and Economics; (2) Others

6. You are _______ (Freshman, Sophomore, Junior, Senior, Master or above)

7. Exempt from tuition fee, your average expenditure in school per month is ________

(1) 0-500 RMB; (2) 500-1000 RMB; (3) 1000-1500 RMB; (4) 1500-2000 RMB; (5) 2000 RMB or above.

9, The annual income of your family is ______

(1) 0-5000 RMB; (2) 5000-10000 RMB; (3) 10000-15000 RMB; (4) 15000-20000 RMB;

(5) 20000-30000 RMB; (6) 30000-50000 RMB; (7) 50000-100000 RMB; (8) 100000 RMB above.

10, Have you got any scholarship in university. ______

1, Yes ; 2,No.

11, How is your score in university.________

1, top ; 2, above average; 3,average; 4,below average; 5, bottom.

12, Do you smoke? ________

1, Yes; 2, No.

13, Have you dated anyone in university. ________

1, Yes; 2, No.

**Survey 2 for All Subjects (Test of Self-Conscious Affect-3)**

Your ID number is______

Below are situations that people are likely to encounter in day-to-day life, followed by several common reactions to those situations. As you read each scenario, try to imagine yourself in that situation. Then indicate how likely you would be to react in each of the ways described. We ask you to rate all responses because people may feel or react more than one way to the same situation, or they may react different ways at different times.

For example:

A. You wake up early one Saturday morning. It is cold and rainy outside.

a) You would telephone a friend to catch up on news.

1---2---3---4---5

not likely very likely

b) You would take the extra time to read the paper.

1---2---3---4---5

not likely very likely

c) You would feel disappointed that it's raining.

1---2---3---4---5

not likely very likely

d) You would wonder why you woke up so early.

1---2---3---4---5

not likely very likely

In the above example, I've rated ALL of the answers by circling a number. I circled a "1" for answer (a) because I wouldn't want to wake up a friend very early on a Saturday morning -- so it's not at all likely that I would do that. I circled a "5" for answer (b) because I almost always read the paper if I have time in the morning (very likely). I circled a "3" for answer (c) because for me it's about half and half. Sometimes I would be disappointed about the rain and sometimes I wouldn't -- it would depend on what I had planned. And I circled a "4" for answer (d) because I would probably wonder why I had awakened so early.

Please do not skip any items -- rate all responses.

1. You make plans to meet a friend for lunch. At 5 o'clock, you realize you

stood him up (you didn’t show up when you said you would).

1. You would think: "I'm inconsiderate."

1---2---3---4---5

not likely very likely

1. You would think: "Well, they'll understand."

1---2---3---4---5

not likely very likely

1. You'd think you should make it up to him as soon as possible.

1---2---3---4---5

not likely very likely

d) You would think: "My boss distracted me just before lunch."

1---2---3---4---5

not likely very likely

2. You break something at work and then hide it.

a) You would think: "This is making me anxious." I need to either fix it or get someone else to."

1---2---3---4---5

not likely very likely

b) You would think about quitting.

1---2---3---4---5

not likely very likely

c) You would think: "A lot of things aren't made very well these days."

1---2---3---4---5

not likely very likely

d) You would think: "It was only an accident."

1---2---3---4---5

not likely very likely

3. You are out with friends one evening, and you're feeling especially witty and attractive. Your best friend's spouse seems to particularly enjoy you company.

a) You would think: "I should have been aware of what my best friend is feeling."

1---2---3---4---5

not likely very likely

b) You would feel happy with your appearance and personality.

1---2---3---4---5

not likely very likely

c) You would feel pleased to have made such a good impression.

1---2---3---4---5

not likely very likely

d) You would think your best friend should pay attention to his/her spouse.

1---2---3---4---5

not likely very likely

e) You would probably avoid eye-contact with everyone for a long time.

1---2---3---4---5

not likely very likely

1. At work, you wait until the last minute to plan a project, and it turns out badly.

a) You would feel incompetent.

1---2---3---4---5

not likely very likely

b) You would think: "There are never enough hours in the day."

1---2---3---4---5

not likely very likely

c) You would feel: "I deserve to be reprimanded for mismanaging the project."

1---2---3---4---5

not likely very likely

d) You would think: "What's done is done."

1---2---3---4---5

not likely very likely

5. You make a mistake at work and find out a-co-worker is blamed for the error.

1. You would think the company did not like the co-worker.

1---2---3---4---5

not likely very likely

1. You would think: "Life is not fair."

1---2---3---4---5

not likely very likely

1. You would keep quiet and avoid the co-worker.

1---2---3---4---5

not likely very likely

d) You would feel unhappy and eager to correct the situation.

1---2---3---4---5

not likely very likely

6. For several days you put off making a difficult phone call. At the last minute you make the call and are able to manipulate the conversation so that all goes well.

a) You would think: "I guess I'm more persuasive than I thought."

1---2---3---4---5

not likely very likely

b) You would regret that you put it off.

1---2---3---4---5

not likely very likely

c) You would feel like a coward.

1---2---3---4---5

not likely very likely

d) You would think: "I did a good job."

1---2---3---4---5

not likely very likely

e) You would think you shouldn't have to make calls you feel pressured into.

1---2---3---4---5

not likely very likely

7. While playing around, you throw a ball and it hits your friend in the face.

a) You would feel inadequate that you can't even throw a ball.

1---2---3---4---5

not likely very likely

b) You would think maybe your friend needs more practice at catching.

1---2---3---4---5

not likely very likely

c) You would think: "It was just an accident."

1---2---3---4---5

not likely very likely

d) You would apologize and make sure your friend feels better.

1---2---3---4---5

not likely very likely

8. You have recently moved away from your family, and everyone has been very helpful. A few times you needed to borrow money, but you paid it back as soon as you could.

a) You would feel immature.

1---2---3---4---5

not likely very likely

b) You-would-think: "I sure ran into some bad luck."

1---2---3---4---5

not likely very likely

c) You would return the favor as quickly as you could.

1---2---3---4---5

not likely very likely

d) You would think: "I am a trustworthy person."

1---2---3---4---5

not likely very likely

e) You would be proud that you repaid your debts.

1---2---3---4---5

not likely very likely

9. You are driving down the road, and you hit a small animal.

a) You would think the animal shouldn't have been on the road.

1---2---3---4---5

not likely very likely

b) You would think: "I’m terrible."

1---2---3---4---5

not likely very likely

c) You would feel: "Well, it was an accident."

1---2---3---4---5

not likely very likely

d) You'd feel bad you hadn't been more alert driving down the road.

1---2---3---4---5

not likely very likely

10. You walk out of an exam thinking you did extremely well. Then you find out you did poorly.

a) You would think: "Well, it's just a test."

1---2---3---4---5

not likely very likely

b) You would think: "The instructor doesn't like me."

1---2---3---4---5

not likely very likely

c) You would think: "I should have studied harder."

1---2---3---4---5

not likely very likely

d) You would feel stupid.

1---2---3---4---5

not likely very likely

11. You and a group of co-workers worked very hard on a project. Your boss

singles you out for a bonus because the project was such a success.

a) You would feel the boss is rather short-sighted.

1---2---3---4---5

not likely very likely

b) You would feel alone and apart from your colleagues.

1---2---3---4---5

not likely very likely

c) You would feel your hard work had paid off.

1---2---3---4---5

not likely very likely

d) You would feel competent and proud of yourself.

1---2---3---4---5

not likely very likely

e) You would feel you should not accept it.

1---2---3---4---5

not likely very likely

12. While out with a group of friends, you make fun of a friend who's not there.

a) You would think: "It was all in fun; it's harmless."

1---2---3---4---5

not likely very likely

b) You would feel small...like a rat.

1---2---3---4---5

not likely very likely

c) You would think that perhaps that friend should have been there to defend himself/herself.

1---2---3---4---5

not likely very likely

d) You would apologize and talk about that person's good points.

1---2---3---4---5

not likely very likely

13. You make a big mistake on an important project at work. People were depending on you, and your boss criticizes you.

a) You would think your boss should have been more clear about what was expected of you.

1---2---3---4---5

not likely very likely

b) You would feel like you wanted to hide.

1---2---3---4---5

not likely very likely

c) You would think: "I should have recognized the problem and done a better job."

1---2---3---4---5

not likely very likely

1. You would think: "Well, nobody's perfect."

1---2---3---4---5

not likely very likely

14. You volunteer to help with the local Special Olympics for handicapped children. It turns out to be frustrating and time-consuming work. You think seriously about quitting, but then you see how happy the kids are.

1. You would feel selfish and you'd think you are basically lazy.

1---2---3---4---5

not likely very likely

b) You would feel you were forced into doing something you did not want to do.

1---2---3---4---5

not likely very likely

c) You would think: "I should be more concerned about people who are less fortunate."

1---2---3---4---5

not likely very likely

d) You would feel great that you had helped others.

1---2---3---4---5

not likely very likely

e) You would feel very satisfied with yourself.

1---2---3---4---5

not likely very likely

15. You are taking care of your friend's dog while they are on vacation and the

dog runs away.

a) You would think, "I am irresponsible and incompetent."

1---2---3---4---5

not likely very likely

1. You would think your friend must not take very good care of their dog or it wouldn't have run away.

1---2---3---4---5

not likely very likely

c) You would vow to be more careful next time.

1---2---3---4---5

not likely very likely

d) You would think your friend could just get a new dog.

1---2---3---4---5

not likely very likely

16. You attend your co-worker's housewarming party and you spill red wine on their new cream-colored carpet, but you think no one notices.

a) You think your co-worker should have expected some accidents at such a big party.

1---2---3---4---5

not likely very likely

b) You would stay late to help clean up the stain after the party.

1---2---3---4---5

not likely very likely

1. You would wish you were anywhere but at the party.

1---2---3---4---5

not likely very likely

d) You would wonder why your co-worker chose to serve red wine with the new light carpet. 1---2---3---4---5

not likely very likely

17. You have contributed more than $10 within the last year to charity.

a) Yes:5

b) No:1
